# RNA-Seq analysis identifies genes associated with differential reproductive success under drought-stress in accessions of wild barley *Hordeum spontaneum*

**DOI:** 10.1186/s12870-015-0528-z

**Published:** 2015-06-09

**Authors:** Sariel Hübner, Abraham B. Korol, Karl J. Schmid

**Affiliations:** Department of Evolutionary and Environmental Biology, University of Haifa, Mt. Carmel 31905, Haifa, Israel; Institute of Plant Breeding, Seed Science and Population Genetics, University of Hohenheim, D-70593 Stuttgart, Germany; Current address: Department of Botany, University of British Columbia, Vancouver, Canada

**Keywords:** Drought tolerance, *Hordeum spontaneum* (wild barley), Reproductive success, Adaptation, RNA-Seq

## Abstract

**Background:**

The evolutionary basis of reproductive success in different environments is of major interest in the study of plant adaptation. Since the reproductive stage is particularly sensitive to drought, genes affecting reproductive success during this stage are key players in the evolution of adaptive mechanisms. We used an ecological genomics approach to investigate the reproductive response of drought-tolerant and sensitive wild barley accessions originating from different habitats in the Levant.

**Results:**

We sequenced mRNA extracted from spikelets at the flowering stage in drought-treated and control plants. The barley genome was used for a reference-guided assembly and differential expression analysis. Our approach enabled to detect biological processes affecting grain production under drought stress. We detected novel candidate genes and differentially expressed alleles associated with drought tolerance. Drought associated genes were shown to be more conserved than non-associated genes, and drought-tolerance genes were found to evolve more rapidly than other drought associated genes.

**Conclusions:**

We show that reproductive success under drought stress is not a habitat-specific trait but a shared physiological adaptation that appeared to evolve recently in the evolutionary history of wild barley. Exploring the genomic basis of reproductive success under stress in crop wild progenitors is expected to have considerable ecological and economical applications.

**Electronic supplementary material:**

The online version of this article (doi:10.1186/s12870-015-0528-z) contains supplementary material, which is available to authorized users.

## Background

Wild barley (*Hordeum spontanuem*) is the direct progenitor of cultivated barley (*Hordeum vulgare*) and the two subspecies do not show a reproductive barrier [1]. Therefore, wild barley was long recognized as a source of useful genetic variation for introgression into modern cultivars to breed more robust varieties that are better adapted to environmental stresses [2–5]. Wild barley occurs in different habitats along the Fertile Crescent including extreme desert environments where it is frequently found in large stands of stable populations [6]. The core region of wild barley is characterized by a wide range of environments with substantial differences between years, especially in the Levant, where Mediterranean and desert climates meet [7].

The ability to survive and reproduce under variable and unfavorable environmental conditions is an important fitness component of individual plants [8]. Measuring Darwinian fitness of single genotypes in natural populations is challenging [9], but can be assessed indirectly by measuring a fitness-associated trait like differential reproductive success (RS) under comparable and controlled conditions [10, 11]. Although the number of offspring does not necessarily reflect success in subsequent generations, it still constitutes a major component of fitness and is of interest for breeding purposes [12]. In plants, RS is affected by overall plant growth and development, but the most sensitive stages to both elevated temperatures and drought are meiosis and early grain maturation [13]. Therefore, the ability to tolerate unfavorable environmental conditions such as drought during reproductive development is a key component of plant RS [14].

The sessile nature of most plant species entails two different strategies to overcome unfavorable environmental conditions: avoidance and tolerance [15]. The avoidance strategy consists of a high growth rate and early flowering time to complete the sensitive reproductive stage before unfavorable environmental conditions decrease reproductive efficiency. This strategy is a major adaptive trait in wild barley [16]. However, early maturation may lead to fewer and smaller grains under cool climatic conditions because sensitive reproductive tissues could be damaged and fertilization may be suppressed [17]. An avoidance strategy is also disadvantageous in years with early drought during the flowering stage, which forces plants to reproduce under unfavorable conditions [18]. Under a large-scale climate change, a pure escape strategy may not be sufficient if environmental change becomes more extreme and variable between years. A second strategy is to tolerate stress by adaptive mechanisms and to continue with reproductive development in spite of unfavorable conditions. This strategy enables the completion of the growing stage and may allow reproduction under a wider range of environmental conditions. It involves different mechanisms like downregulation of metabolism, partitioning of amphiphilic compounds and immobilization of cytoplasm, which may vary according to the level of dehydration and maintain sustainable populations at periods of adverse conditions [19]. In wild barley, both fecundity and maternal investment are sensitive to environmental changes and subject to natural selection (e.g., [20]). Therefore, plants that reproduce in adjacent years with similar or different environmental conditions are under constant selection, which enhances adaptation to fluctuating environments [8]. Both the escape and tolerance strategies are adaptive responses to environmental selective pressures and may coexist in a population, but the tolerance strategy helps to maintain stable populations over time and is of interest for breeding varieties with a higher yield stability in changing environments [21].

The identification of reproductive drought-tolerance genes is essential for understanding the molecular mechanisms of drought tolerance and plant adaptation. One approach to identify such genes is to compare transcript levels at the reproductive stages among drought-tolerant and sensitive accessions that were exposed to drought treatment [22]. A differential expression analysis to detect drought-tolerance genes in the wild ancestor of a major crop may contribute to a better understanding of RS mechanisms and the utilization of beneficial alleles for breeding of more robust varieties.

Expression profiling by massively parallel cDNA sequencing (RNA-Seq; [23]) is a cost-effective way to survey transcriptomes of different tissues and developmental stages. RNA-Seq accurately identifies gene expression profiles [24] with an appropriate experimental design, and may not require a validation step with another method such as quantitative PCR [25, 26]. Thus, RNA-Seq is becoming the technology of choice for studying expression profiles of non-model organisms [27, 28]. Since RNA-Seq enables to combine gene discovery with the identification of allelic variation, sequence variants associated with differentially expressed genes in response to a treatment can be identified. Such trait-associated variants are of prime interest for applying marker-assisted selection in advanced breeding programs [29].

In this study, we addressed three objectives: (i) to phenotypically discriminate between drought-tolerant and sensitive accessions with respect to RS under terminal drought stress, (ii) to detect differentially expressed genes associated with drought tolerance, and (iii) to investigate ecological and evolutionary aspects of drought responsive genes in wild barley originating from different eco-geographical regions in Israel. We applied RNA-Seq to drought-tolerant and sensitive wild barley accessions grown in a common-garden experiment to study the genomic basis of RS under drought and identified numerous candidate genes involved in the response to drought during reproductive development. This study provides ecological and evolutionary insights into plant adaptation and an applied perspective for crop breeding.

## Results

### Selection of drought-tolerant and sensitive accessions

**Fig. 1:**
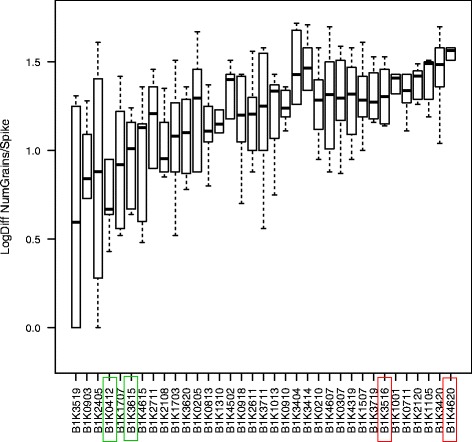
Number of grains lost by the drought treatment for each of the 35 accessions in the drought experiment conducted at the Aaronson farm in 2010. The dark horizontal line indicates the median, boxes represent the range between first and third quartiles and whiskers extend to the extremes. Tolerant accessions are marked with a green and sensitive accessions with a red box

The Barley1K collection consists of wild barley ecotypes representing the wide eco-geographical diversity in the Southwestern part of the Fertile Crescent [30]. The 35 selected accessions reflect the different eco-geographical regions and phenological ecotypes present in this collection [31]. We previously verified that all accessions used in this study are free of traces of recent introgressions from cultivated barley [32]. A common-garden experiment with these accessions was conducted in a greenhouse during the winter of 2010 to evaluate their reproductive success under terminal drought treatment (Fig. 1, Additional file 1: Figure S1 A,B). We define reproductive success as relative grain loss between treated and untreated plants due to water deficit during flowering and early maturation. The standard deviation of relative grain loss correlated with mean grain number (*r*^*2*^ = 0.24, *p* = 0.004). To reduce this scaling effect we transformed the calculated difference between drought and control treatments within blocks to a logarithmic scale (*r*^*2*^ = 0.19, *p* = 0.01). Since the extent of grain loss due to drought indicates the ability of a plant to reproduce despite unfavorable drought conditions, we considered smaller differences between treatments as higher drought tolerance. We first tested whether RS in the first year experiment was correlated with eco-geographic gradients in the native distribution range [30]. Mean grain loss in response to drought did not correlate with level of precipitation (Pearson’s *r* = −0.19, *p* = 0.26), geographic distance (*r* = −0.06, *p* = 0.12) or genetic distance calculated from 42 microsatellite markers [30],[32] (*r* = 0.04, *p* = 0.3). The latter result indicates that differences in drought tolerance do not result from genetic drift and population structure.

**Fig. 2:**
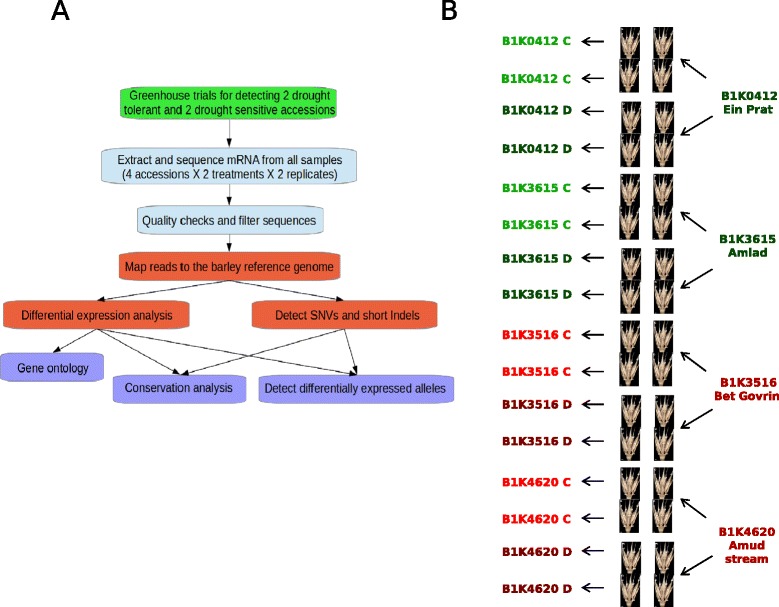
General workflow of the study. **a** Analysis of differentially expressed genes associated with drought tolerance from greenhouse trials to candidate genes. **b** Sampling strategy to produce the 16 sequenced cDNA libraries. For each tolerant (*green*) and sensitive (*red*) accessions two pooled spikelets were sampled at two replicates for each drought (*dark*) and control (*light*) treatments for RNA extraction and sequencing

Based on the common-garden experiment, we selected two tolerant and two sensitive accessions for further analysis (Table 1, Fig. 2) and repeated the experiment with the same design in the following year under controlled conditions in a greenhouse at the University of Hohenheim, Germany (Additional file 1: Figure S1 C,D). A two-way ANOVA was performed with the four accessions to test the effect of drought treatment applied to the selected accessions in the two environments (Atlit and Hohenheim) on the number of grains per spike. The treatment (drought vs. control) caused strong differences in grain number (MS = 802.4, *F* = 44.09, p < 10^−7^), while the environment (MS = 4.7, *F* = 0.26, *p* = 0.61) and treatment × environment interactions (MS = 0.7, *F* = 0.04, p = 0.84) had no effect, which may reflect the controlled conditions of the experiment. A further two-way ANOVA quantified the effects of the environment and a classification into drought-tolerant or sensitive accessions on the extent of grain loss in response to the drought treatment. The two tolerant accessions differed significantly from the sensitive accessions in the number of grain loss (MS = 0.87, F = 25.73, *p* = 5.28 × 10^−5^) regardless of the environmental effect or the classification × environment interaction (MS = 0, F = 0, *p* = 0.99 and MS = 0.04, F = 1.24, *p* = 0.28, respectively), which confirmed the results of the first year experiment.

**Table 1:** The four wild barley accessions selected from the Barley1K collection for drought response screening and expression profiling after the phenotypic greenhouse trials in Atlit and Hohenheim

Accession ID	Response	Sampling site	Coordinates	Mean days to heading (control/drought)	**∆**Log Grains/Spike 2010 (sd)
B1K0412	Tolerant	Ein Prat	353056E, 318346 N	92/95.8	0.88 (0.50)
B1K3615	Tolerant	Amiad	355318E, 329259 N	101/99	1.01 (0.15)
B1K3516	Sensitive	Beit Govrin	348992E, 315952 N	100/105	1.31 (0.16)
B1K4620	Sensitive	Amud stream	355028E, 328723 N	102/99	1.58 (0.09)
All accessions				101.3/101.4	1.17 (0.38)

Days to heading and difference (∆log) in number of seeds/spike are indicated for the 2010 experiment

### RNA-Seq and reference transcript assembly

In the second-year experiment (U. Hohenheim), we sampled spikelets at the fertilization stage from each accession under treatment and control conditions for further analysis. Sampled mRNA was sequenced (RNA-Seq) to identify candidate genes that are associated with differential reproductive success under drought stress. We pooled the two spikelets to create a single sample for each of two tillers taken from each of eight plants (4 genotypes x 2 treatments) during flowering stage (Fig. 2). We extracted total RNA from each of the 16 samples and obtained 80 Gb of poly(A)-selected RNA sequences with an average of 50 million single-end reads with lengths of 96 bp per library (Additional file 2: Table S1). The preprocessing step removed 16-19 % of reads per library due to low quality. Read mapping to the Morex reference assembly with TopHat and Cufflinks produced assemblies of more than 40 million reads per library with an average of 70x per-base coverage. These assemblies correspond to a total of 189,919 isoforms including 95,387 multi-exon transcripts and 20,905 multi-transcript loci with approximately 2.1 transcripts per locus over all accessions. Integrating the reference annotation file in the transcript assembly pipeline identified 49,340 coding sequences including novel transcripts in each accession. Among all libraries, 6.8-9.8 % of assembled loci, 5.2-6.5 % of exons and 6.3-7.4 % of introns were novel with respect to the reference genome. We further analyzed the assemblies with SAMTools to call SNPs and short indels in the four wild barley accessions. Altogether, 298,778 SNPs and short indels with a phred-quality >20 were identified. Interestingly, more polymorphisms segregated in the two Northern (mean = 229,828) than in the Southern accessions (mean = 226,224) regardless of the number of isoforms in each accession (*r* = 0.1; *p* = 0.9). This may reflect a higher level of genetic variation in Northern ecotypes. Additional alignment information for each accession is given in Table 2.

**Table 2:** Summary of transcript assemblies and annotations for each of the four accessions analyzed

Accession ID	Average number of aligned reads (sd)	Average coverage (sd)	Isoforms	SNPs	Introns	Exons
B1K0412	47,259,206 (5,881,069)	69.96 (11.16)	152,061	227,441	149,604	323,806
B1K3516	51,879,703 (14,491,956)	66.83 (10.55)	156,474	225,007	151,973	330,710
B1K3615	53,149,174 (13,822,620)	78.18 (16.11)	156,951	230,152	151,794	330,838
B1K4620	45,748,092 (11,603,178)	70.01 (6.08)	155,644	229,505	151,416	329,419

For each accession the average number of aligned reads and coverage from the corresponding four libraries (two drought and two control) and standard deviations are indicated

### Differential RNA expression in drought tolerant and sensitive accessions

**Fig. 3:**
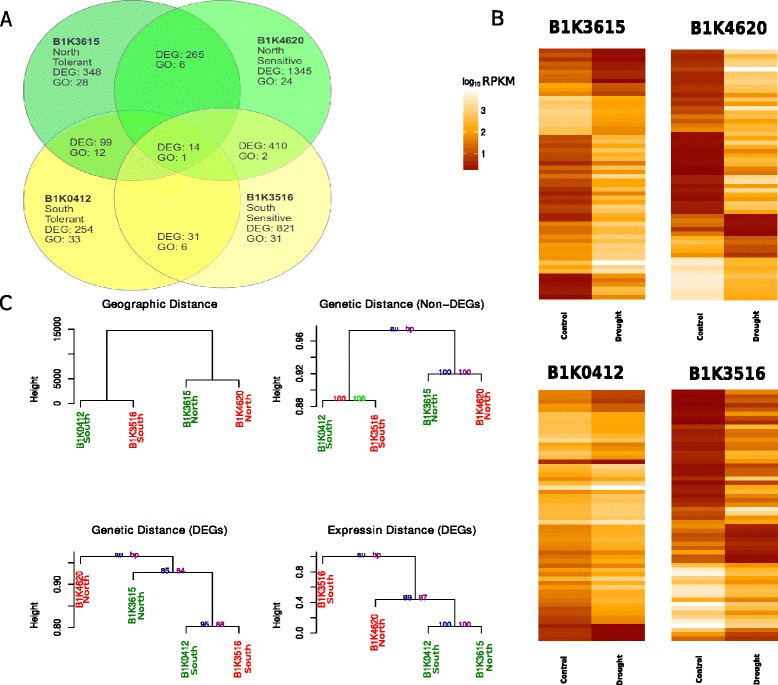
Analysis of differential expression in ‘drought’ versus ‘control’ treatments. **a** Venn diagram of overall differentially expressed genes and the corresponding number of significantly enriched (FDR < 0.05) gene ontology in response to drought treatment. **b** The top 50 significant differentially expressed genes between drought and control treatments for each accession. **c** Dendrograms of geographic distances between accessions, genetic distances based on differentially (DEGs) and non-differentially expressed genes (Non-DEGs), and expression distance calculated from the log-fold change in differentially expressed genes (DEGs). Drought tolerant accessions are printed in green and sensitive accessions in red and their region of origin (north/south) is indicated below. Bootstrap probability values (bp) are printed in purple and approximate unbiased probability (au) values are printed in blue

To identify genes associated with response to drought stress in tolerant and sensitive accessions, we quantified gene expression in spikelets sampled at the early flowering stage (see ‘Materials and Methods’). The experimental design and replicated sampling allowed us to control for residual variation within each accession (Fig. 2). To test whether genetic drift (isolation-by-distance effects) and physiological adaptation contribute to expression differences, we compared expression, genetic, and geographic distances between accessions. The two Northern accessions had approximately six times more differentially expressed genes in common than the Southern accessions after correcting for the total number of genes (Fig. 3a). Among the top 50 differentially expressed genes in each accession (Fig. 3b), 8 genes were shared among sensitive accessions, 15 among tolerant accessions, none among southern accessions, 12 among northern accessions and none among all accessions. Hierarchical clustering of SNPs in genes that are constitutively expressed across accessions grouped the four individuals in accordance with their geographic origin (north/south) as expected by the effect of neutral genetic drift (Fig. 3c). However, the same clustering analysis based on SNPs in genes that were differentially expressed in drought-tolerant accessions but not in drought-sensitive accessions (drought-tolerance genes) resulted in a weaker geographic clustering as indicated by the corresponding bootstrap values (Fig. 3c). Moreover, clustering the accessions based on their expression profiles grouped the accessions in accordance with their phenotypic drought-response classification (tolerant/sensitive) rather than their geographic origin and showed that the expression of drought response genes is not consistent with their eco-geographic origin (i.e., Mediterranean vs. desert climate). In addition, we found no correlation between geographic and genetic distances calculated from 723 SNPs detected in drought-tolerance genes (*r* = −0.06, *p* = 0.91), nor between genetic distance and differential expression profiles of these genes (*r* = −0.36, *p* = 0.47). Taken together, the results indicate that the drought-response phenotype and the associated transcriptome patterns are not associated with a putative local adaptation to major habitats (Mediterranean vs. desert) but represent a polymorphic physiological response mechanism.

**Fig. 4:**
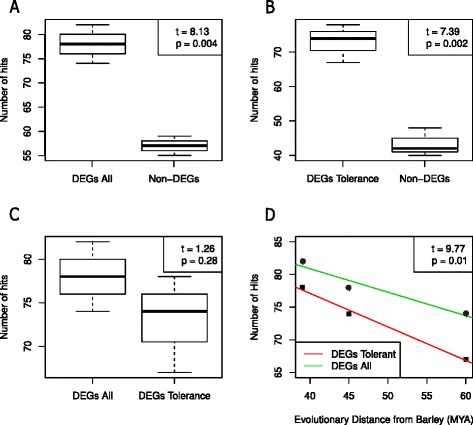
Evolutionary conservation based on the proportion of BLAST hits to *Brachypodium distachyon*, *Oryza sativa,* and *Sorghum bicolor* non-redundant protein databases. The dark horizontal line indicates the median, boxes represent the range between first and third quartiles and whiskers extend to the extremes. For each comparison A-D, *t* scores and *p*-values are indicated in top-right box. **a** drought-responsive genes in all accessions (DEGs All) versus non-responsive genes (Non-DEGs), **b** drought-tolerance genes (DEGs Tolerance) versus non-responsive genes, **c** The drought-responsive genes in all accessions versus drought-tolerance genes, and **d** drought-responsive genes in all accessions (*green*) versus drought-tolerance genes (*red*) conservation along time scale since divergence from barley

To test the hypothesis that the genetic basis of drought tolerance represents a recent adaptation, we compared genetic diversity and the long-term evolutionary conservation of genes representing the three major types of expression patterns: constitutively expressed in all accessions, drought-responsive (differentially expressed in all accessions), and drought-tolerant (differentially expressed in drought-tolerant and constitutively expressed in drought-sensitive accessions). We selected a random set of 50 genes from each group to achieve balance between representation and randomness with respect to the total number of genes in each of the three groups (see Fig. 3a). The number of SNPs was used as a measurement for genetic diversity after correcting for transcript length. Constitutively expressed genes showed a lower diversity (4.51 SNPs/Kb) than both drought-tolerance (6.63 SNPs/Kb, t_welch_ = 2.26, *p* = 0.02) and drought-responsive genes (6.18 SNPs/Kb, t_welch_ = 2.67, *p* = 0.008). The average diversity of drought-tolerance genes was not significantly higher than of drought-responsive genes (t_welch_ = 0.23, *p* = 0.82). To characterize the evolutionary conservation of drought-responsive compared with non-responsive genes we randomly sampled 100 genes showing differential or non-differential expression in response to the drought treatment separately for each accession (Fig. 4). We determined the level of conservation by sequence comparison to homologs in *Brachypodium distachyon*, *Oryza sativa,* and *Sorghum bicolor* using the sequences from the barley reference assembly (Morex) as query sequence to reduce any mismatch effect resulting from sequence diversity in the wild barley accessions. The group of drought-responsive genes (differentially expressed in all accessions) showed more hits against the three species than non-responsive genes (*t*_Welch_,= 8.13, *p* = 0.004) indicating that drought-responsive genes tend to be more conserved. The drought-responsive genes were also more conserved than drought-tolerance genes, which are differentially expressed only in drought tolerant accessions (*t* = 9.77, *p* = 0.01; Fig, 4d).

Fourteen genes were differentially expressed in response to drought treatment across all accessions, of which 12 genes are associated with drought stress (e.g., Paired amphipathic helix protein LEA [33], expansin [34] and VQ-motif transcription factor [35]; Additional file 3: Table S2). Overall, more differentially expressed genes were found in the sensitive (B1K4620 = 1,345, B1K3516 = 821) than in the tolerant accessions (B1K3615 = 348, B1K0412 = 254). Out of 99 differentially expressed genes in drought tolerant accessions, 85 were detected only in the two tolerant accessions and not in the sensitive accessions of which 6 are drought-associated transcription factors (e.g., WRKY, BZIP, MADS-Box), 5 unclassified retrotransposon proteins and transposase, 5 fertility-associated genes (e.g., *Chalcone synthase*, *Squalene synthase,* and *Prostaglandin E synthase*), and 13 genes of unknown function. We consider these as candidate genes that contribute to reproductive success under drought stress in wild barley (Additional file 4: Table S3). In addition, 396 genes were differentially expressed in the drought-sensitive but not in the tolerant accessions. Among these genes, several drought-induced genes were detected (e.g., AP2, U-box, *Serine proteases*, and *Peroxidase*), and 50 genes of unknown function, which are candidate genes for further studies (Additional file 5: Table S4).

### Functional annotation of differentially expressed allels

**Fig. 5:**
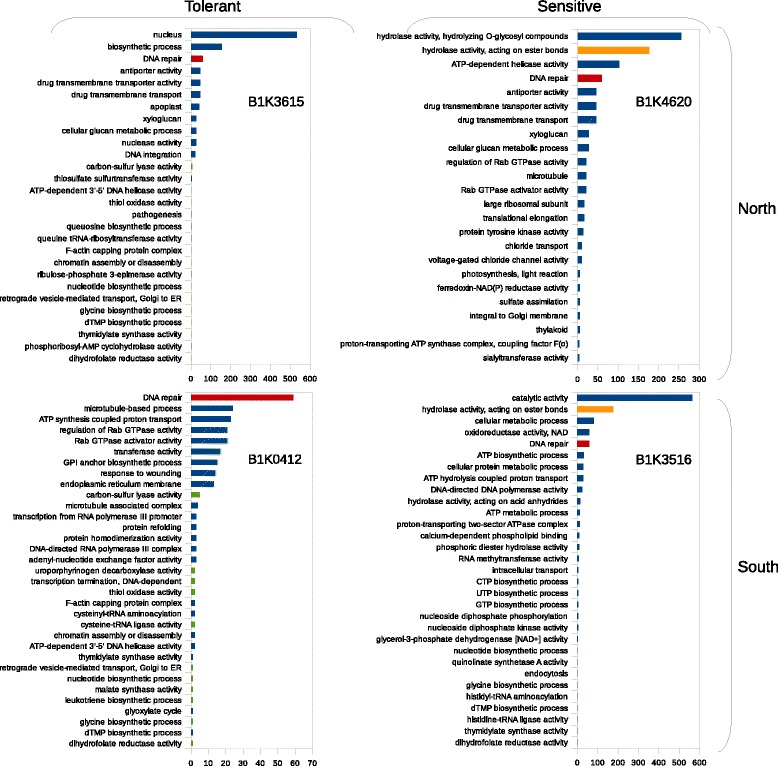
Functional annotation analysis of overall gene expression. Distribution of significantly enriched GOs in all accessions. Shared category among all accessions is colored with red, shared categories among tolerant accessions is colored in green, and shared categories among sensitive accessions is colored in orange

To infer the biological processes and functions of genes associated with drought stress response, we conducted a gene ontology (GO) analysis separately for each accession (Additional file 6: Table S5). Although more genes were differentially expressed in sensitive (410) than tolerant accessions (99), more GO categories were enriched in tolerant compared with sensitive accessions. Altogether, 90 categories were enriched (FDR < 0.05) in all samples, and DNA repair was the only category enriched across all accessions. Two categories (hydrolase activity and DNA repair) were enriched in the sensitive accessions and 12 categories (e.g., DNA helicase activity, thiol oxidase activity, and glycine biosynthetic process) in the tolerant accessions (Figs. 3a and 5). Of the 12 categories enriched in the tolerant accessions, at least four categories are associated with carbon metabolism, which has an important role in enhanced stress tolerance in plants.

We further investigated sequence variation in drought-tolerance genes. The 99 genes differentially expressed in the tolerant accessions harbor 1,056 high quality (phred score > 20) SNPs and short indels, of which 42 polymorphisms differentiate between the two drought-tolerant and the sensitive accessions. To examine whether alleles that are specific to drought-tolerant accessions and different from the Morex reference are a potential source of useful genetic variation, we selected four candidate drought-tolerance genes and characterized potential functional effects of allelic variation with the SnpEff program (Table 3, Additional file 7: Figure S2). Two genes were previously associated with drought response (AK362742, AK368692), one with pollen viability (MLOC_67950.1), and one is of unknown function (AK370720). In AK368692, one variant was located upstream to the coding region and in AK362742 two variants were synonymous substitutions. In MLOC_67950.1, a non-synonymous substitution (GtG/GcG: valine/alanine) was found in the coding region and one allele in AK370720 was detected (aAG/tAG: Lysine/stop) as leading to premature stop codon.

**Table 3:** Differentially expressed alleles between tolerant and sensitive accessions

Contig	Gene ID	Gene Description	POS	Chr	REF	ALT	QUAL	Effect	B1K0412	B1K3516	B1K3615	B1K4620
morex_contig_53956	MLOC_67950.1	GPI mannosyltransferase	7720	5HL	A	G	243	Moderate	1/1	0/0	1/1	0/0
morex_contig_65282	AK362742	Dehydrogenase/ reductase	3352	6HS	C	T	28.6	Low	1/1	0/0	1/1	0/0
morex_contig_1575714	AK368692	Vacuolar protein	4665	6HL	C	T	80.4	Modifier	1/1	0/0	1/1	0/0
morex_contig_1734244	AK370720	unknown protein	82	7HL	A	T	48.8	High	1/1	0/0	1/1	0/0

Candidate variants after filtering for heterozygosity and including only non-reference alleles associated with drought tolerance. The contig name in the Morex assembly is indicated, the annotated gene (Gene ID), variant position within contig (POS), chromosome arm (Chr), the reference (REF) and alternative (ALT) variants, the phred-quality (QUAL), the variant impact (Effect), and the genotype of each accession

## Discussion

In this study, we combined phenotypic analysis with RNA-Seq to investigate the phenotypic variation and transcriptomic basis of reproductive success under drought stress in the wild ancestor of cultivated barley. We observed a substantial level of phenotypic variation among accessions and found that gene expression patterns are similar between drought tolerant accessions with different genetic background and geographic origin.

### Detection of drought-tolerant accessions from natural populations

The 35 accessions selected from the Barley1K collection for this study represent the three major ecotypes in the Levant [30–32]. These accessions were screened to test whether they differ in reproductive success under terminal drought stress in controlled conditions. We further verified that the four selected accessions for the expression analysis truly represent differences in response to drought and that our drought-treatments was the major contributor to reduction in the number of seeds for each accession. Both tests confirmed our experimental setup and indicated a marginal contribution of the environment and environment × genotype interactions. Although isolating the factors of interest is the major benefit of a common-garden experiment, the response to drought under natural conditions is an ensemble of interactions with many abiotic and biotic factors involved. The comparison of the relative number of grains produced under drought with geographic distance, genetic distance, and precipitation gradient revealed different levels of reproductive success within ecotypes regardless of their eco-geographic location. This result contrasts previous studies [36] and suggests that reproductive drought tolerance is not solely restricted to areas of low precipitation, which is consistent with the hypothesis that alternating selection (e.g., through changing the physiological optimum) may act to maintain a population under changing environmental conditions [37]. Therefore, accessions with high reproductive success under unfavorable environmental conditions are expected to occur also in regions with changing precipitation in adjacent years. Clustering of expression data further supported our observation that similar physiological responses are found in different ecotypes (desert vs. Mediterranean). In contrast, the SNPs identified in the transcriptome sequences clearly grouped the four accessions in accordance with their eco-geographic origin, thereby supporting the previous population genetic and phenotypic analyses of the Barley1K collection [30–32]. The clustering analysis with SNPs from drought-tolerance associated genes differentiated the ecotypes less, which suggests that these genes evolved differently than other parts of the transcriptome. Although genetic drift leads to genomic divergence in accordance with isolation by distance, physiological adaptation to similar types of stress in different regions may occur through a small number of genetic changes, which influences the clustering mode. Additional causes for the differential response to drought and the underlying gene expression may involve changes in gene regulation by structural variation [38], epigenetic modifications of chromatin state [39], transposable elements activity [40], or a combination of more than one mechanism.

Drought is a major selective constraint in the evolution of plants. However, the relative contribution of selection acting on new and standing genetic variation, or phenotypic plasticity is still unknown. Although drought is seen as a diversifying factor in population dynamics [30] we show that in contrast to previous studies [41] and in accordance to others [42, 43], a substantial variation exist within diverged populations in drought response, supported by both phenotypic and transcriptome analysis. Further analysis is required to quantify the relative contribution of adaptive phenotypic plasticity and pleiotropic gene action to drought tolerance in plants [44] as well as the role of genetic and epigenetic factors.

### Evolution of drought-tolerance genes

The differential gene expression in plants under drought and control treatments for both tolerant and sensitive accessions enabled us to identify sets of genes associated with reproductive success under terminal drought in accessions from different eco-geographical regions. Drought-responsive genes common to all accessions are more evolutionarily conserved than non-differentially expressed genes. High evolutionary conservation is expected for functionally important genes due to purifying selection that reduces the rate of evolution relative to neutrality [45, 46]. In genes associated with an avoidance-strategy like flowering time variation, different alleles may be fixed along eco-geographic gradients [16], whereas drought-tolerance genes are expected to evolve under balancing selection in different geographic regions [47]. Our results support this observation because of a higher genetic diversity in drought-responsive than non-responsive genes. In addition, genes associated with drought-tolerance, which are differentially expressed only in tolerant accessions, tend to evolve faster than other drought-responsive genes (differentially expressed in all accessions). Relative position in the signaling pathway associated with the response to drought may be a plausible explanation in linear biochemical networks [48]. However, in more complex networks (as in our case) the correlation between function and rate of evolution is less obvious.

### The genetic basis of reproductive success under drought in wild barley

Drought stress during reproductive stages may reduce yield by up to 60 %, mostly due to reduction in grain number [49]. The traits most sensitive to reproduction-associated drought stress are pollen viability, stigma receptivity, panicle exertion, anther dehiscence, and early grain development [13]. We found differentially expressed genes associated with these traits in this study ( Additional file 3: Table S2). The most prominent biological process enriched in all accessions in response to drought was DNA repair which plays a critical role during meiosis [50] and seed development [51]. Several genes associated with reproductive success under stress were detected exclusively among the drought-tolerant accessions, and could potentially be used for breeding of more drought tolerant varieties ( Additional file 4: Table S3). For example, the flavonoid synthesis pathway gene *Chalcone synthase* was identified among the candidates (Log-fold change = 2.87; Additional file 4: Table S3). Although its mechanistic role in drought stress is still unknown, *Chalcone synthase* was previously reported as a contributor to reproductive success under heat stress [52]. Another group of genes associated with drought tolerance were bZIP transcription factors that are involved in both response to stress and reproductive development success [53]. Several genes associated with response to drought stress were detected among the drought-sensitive accessions. Interestingly, AP2 of the super-family of DREB genes was found among the over-expressed genes in response to drought. The DREB protein family comprises important plant transcription factors that regulate the expression of numerous stress-responsive genes, and DREB proteins associated with enhanced stress tolerance [54]. A possible explanation for the higher expression of DREB proteins among sensitive than tolerant accessions is that the drought-tolerance mechanisms during the vegetative state (in which AP2 is expressed) is different from the mechanism acting during fertilization and reproduction [12]. The adaptive value of genes expressed in sensitive accessions is unknown and requires further study.

Among the tolerant accessions, several categories associated with carbon metabolism were enriched. Drought stress can affect plant viability through carbon starvation, which is tightly interdependent on both the avoidance and occurrence of hydraulic failure through impacts on maintenance metabolism [55]. An increased carbohydrate content threshold in tolerant accessions is a possible mechanism by which increased fitness under drought stress is achieved. Another enriched process associated with drought tolerance involves thiol metabolism (three enriched categories), which is a central mechanism of protecting plants from oxidative damage caused by environmental stresses such as drought [56]. To better understand the contribution of these biological functions to drought tolerance further support is needed from metabolic pathways analysis and eQTL mapping in a segregating population [57].

One advantage of RNA-Seq is the combination of differential expression analysis with sequence polymorphism detection, which allows to associate differentially expressed alleles with a trait of interest and to identify potential effects on protein function [58, 59]. In this study, we predicted the expected effect of differentially expressed alleles in protein function in four candidate genes after filtering for low quality SNPs [60]. Three of these candidate genes were identified as drought responsive genes and one is of unknown function. These genes are known to be associated with pollen viability, ABA biosynthesis [61], and vacuolar processes, which contribute to an increased flexibility to cope with environmental changes [62]. These genes are major candidates for increasing RS under drought stress and can serve as a lead for further functional and physiological studies to unravel the complex mechanisms associated with drought tolerance in plants. It should be noted that SNP annotations and effect predictions need to be addressed with caution because they rely on robust sequence annotation which is still under development for barley.

Here we showed that the differential response to drought stress of tolerant and sensitive plants during reproductive development is the outcome of adaptation to common environmental stress regardless of eco-geographic and genetic distances. Reproductive success in wild barley under drought stress is not an ecotype-specific trait that evolved as a local adaptation, but appears to be a physiological adaptation which evolved similarly in different regions and which is characterized by an increased evolutionary flexibility.

## Conclusions

Reproductive success under drought stress is an important trait in the study of fitness and adaptation in natural populations and for breeding high yielding varieties that can sustain harsh environments. Using transcriptome analysis of a common-garden experiment we show that reproductive success under drought stress has evolved similarly in different habitats indicating a shared physiological adaptation. Moreover, drought responsive genes were found to be more conserved in evolution than non-responsive genes and drought-tolerant genes were found to evolve recently in the evolutionary history of wild barley.

## Methods

### Plant material and field trials

We selected representative wild barley accessions from different eco-geographical regions in the southwestern part of the Fertile Crescent from the Barley1K collection [30]. These accessions were grown in a greenhouse under common-garden conditions in the winter of 2010 at the Aaronson farm in Atlit, Israel (32°42’35”N, 45°33’34”E). Each of the selected 35 accessions was sterilized with 4 % NaOH, wrapped in germination paper, and placed in a cold dark room (4 °C) for one week to break dormancy and improve germination. Germinating seeds were transplanted into 5-l pots containing a sand and pit mixture (1:1) and placed in a greenhouse under full irrigation regime with a daily amount of 200 cm^3^ water per plant. The experiment was conducted in a randomized block design (RBD) where both ‘drought’ and ‘control’ treatments for each accession were replicated once within each of six blocks. This experimental design enabled to evaluate the differences between treatments with the lowest experimental variation.

All plants were irrigated daily using a drip system until the flag leaf emerged in 90 % of the plants. The drought treatment was implemented for half of the plants by preventing watering for 12 days, until tensiometers placed into pots indicated that one-third of the field capacity and the wilting point (yellow leaves) had been reached. Plants under the drought treatment were then irrigated with 200 cm^3^ water per plant every fourth day to maintain the stress until all plants were harvested. During this time, a full irrigation regime was implemented to control plants. In each plant, five spikes were covered to avoid grain loss from seed shattering. The total number of filled seeds was counted for each plant, and the difference between control and drought treatments within each block was calculated as a measurement for the reproductive response to drought. Reproductive response measurements were then transformed to a logarithmic scale due to standard deviation-mean dependence for the number of grain loss i.e., standard deviation increase with average number of seeds [63]. In the following year, the experiment was repeated with selected accessions in a greenhouse at the University of Hohenheim, Germany (48°71'38''N, 9°20'88''E). The same experimental procedure was conducted in three replicates and compared to the results from previous year. Two tolerant and two sensitive accessions were selected for mRNA extraction in the second year conducted in Hohenheim based on their performance in the first year experiment i.e., the average reduction in seed set in response to drought with minor spread of data (see Figs. 1 and 2). From each plant, two spikelets were sampled from the middle of two different spikes at the early flowering stage (booting, Zadok scale: Z49; [64]) when fertilization occurs and stress has the strongest effect on yield reduction [65].

### RNA extraction and sequencing

Samples were immediately frozen in liquid nitrogen and stored in −80 °C until RNA extraction. Total RNA was extracted from 16 samples (Fig. 2) using the Bioline Mini Kit protocol for plants (Bioline GmbH, Germany). An amount of 1.5-10 μg was determined using an electrophotometer with an OD 260/280 ratio of at least 1.8 and gel electrophoresis. Quality was determined by using a RNA integrity number (RIN) of at least 8. Extracted RNA samples were further processed by SciLifeLab, Stockholm (School of Biotechnology, KTH) and included the purification of polyadenylated RNA, RNA fragmentation, cDNA synthesis, polymerase chain reaction (PCR) amplification, and RNA sequencing according to the Illumina RNA-Seq protocol (Illumina, Inc., San Diego, CA). Single read non-strand-specific RNA sequences (RNA-Seq) were generated using a HiSeq2000 in one flow cell with eight lanes producing 50 million reads of 100 bp per sample. Each of the 16 libraries was tagged and split between two lanes. Within each lane, random combinations of four different half libraries were injected to reach a balanced experimental design [66] and to reduce sequencing bias resulting from differences in sample amplification efficiency of different libraries.

### Identification of differentially expressed transcripts

Sequenced Illumina reads were mapped to the annotated barley genome assembly of the Morex variety [67] using Bowtie2 v2.0.5 [68] and TopHat v2.0.6 [69]. Each of the 16 transcriptome libraries was separately mapped to the reference genome after trimming tags and filtering low-quality reads based on the distribution of phred-like scores at each sequencing cycle with the FASTX toolkit (hannonlab.cshl.edu/fastx_toolkit/) using the command: fastq_quality_filter -v -q 20 -p 80. TopHat was run with default parameters and a gene model annotation file in the GTF format was used to enable Bowtie2 to first align transcript sequences to the transcriptome and then to map only unmapped reads to the genome.

To measure the level of expression, the quantification of transcript abundance in the samples was conducted with Cufflinks v2.0.2 [70]. Assemblies were produced separately for each of the 16 libraries and then parsimoniously merged with the reference genome annotation for each accession (four libraries each) using *Cuffmerge*. This step performs an assembly based on the annotation to the reference [70] and produces an annotation file for downstream analysis. Since annotation files were generated separately for each accession, an additional step matched transcripts from different samples and produced an annotation file that combined the transcribed fragments (transfrags) from all accessions and the reference annotation file. This step was carried out with *Cuffcompare* and utilized the reference genome annotation to produce a single annotation file for downstream analysis of differential expression. Changes in the relative abundance of transcripts between drought treatment and controls were estimated for each accession using the *Cuffdiff* program, which calculates the number of reads per kilobase of exon per million reads mapped (RPKM) for each transcript and summarizes them for each group of transcripts [23]. Cuffdiff output files were further processed with the R package *CummeRbund* [71] to integrate the output from different accessions into a single data set for differential expression analysis and visualization. Differentially expressed genes were considered significant at log_*e*_ of fold change of 1.5 and FDR level of 0.05 using the Benjamini-Hochberg method [72].

Clustering of genes classified as differentially expressed was conducted with the *pvclust* package in R [73]. Expression distances among accessions were calculated from the log_*e*_ of fold change between treatments. Genetic distances were calculated from single nucleotide polymorphisms (SNPs) using the pair-wise Hamming distance between accessions. Geographic distances were calculated using the pair-wise Euclidean distance based on the Barley1K sampling site coordinates. Clustering trees were bootstrapped 1,000 times to evaluate the robustness of clustered nodes.

### Evolutionary sequence conservation

To assess the evolutionary conservation of genes associated with the response to drought, a sample of 100 genes that were differentially expressed between drought and control, and 100 non-differentially expressed genes were randomly sampled from each accession to balance between good representation of the data sets and randomness. This sub-sampling step was conducted to avoid a bias introduced by differences in sample size between the two data sets. For each gene, the corresponding sequences were retrieved from the reference genome to reduce the effect of inter-accession polymorphism on the cross-species mapping success. Sequences were mapped to protein databases of *Brachypodium distachyon*, *Oryza sativa,* and *Sorghum bicolor* (http://www.plantgdb.org/) using BLASTX [74] with an E-value cutoff of 10^−3^, and the number of hits and best hit E-values were recorded.

### Gene ontology analysis

To study the biological significance of differentially expressed genes, an enrichment analysis of gene ontology (GO) terms was conducted with the Bioconductor package *goseq* [75], which accounts for biases inherent in RNA-Seq data*.* Statistically significant over-representation of GO categories in response to the drought treatment was determined separately for each accession. A list of differentially expressed genes, after correcting for multiple comparisons (adjusted p-value < 0.05), and of non-differentially expressed genes was used to generate the probability weighting function which enables to correct for the gene coding length bias. To save computing power, the Wallenius distribution method [75] was used as an approximation for the null distribution. The length of each transcribed gene was calculated from its coordinates in the reference genome and a corresponding category mapping file was generated using available annotation database (ftp://ftpmips.helmholtz-muenchen.de/plants/barley/public_data/). Significantly over-represented GO terms were finally corrected for multiple comparisons at FDR level of 0.05 using the Benjamini-Hochberg method [72].

### Identification of SNPs

To study differentially expressed alleles that are associated with drought tolerance, single nucleotide polymorphisms (SNPs) were detected in each accession by comparing the transcript sequences to the Morex reference assembly. For each accession, all four libraries were concatenated after removing low-quality sequences to generate the deepest and widest possible transcriptome representation. Each of the four concatenated files was mapped to the Morex assembly using Bowtie2. Variant calling was conducted using the *mpileup* function in SAMTools v1.18 [76] and the BCFTools package was used to filter SNPs with a minimum phred quality of 20 and a maximum read depth of 100. Heterozygote SNPs, which could be the outcome of sequencing errors, collapsed paralogous genes or remnants of a past introgression were filtered out as well. Candidate variants from differentially expressed genes were further filtered and their functional annotation was predicted with SnpEff [60].

### Availability of supporting data

Sequence reads were submitted to the European Nucleotide Archive under accession number PRJEB8700. All aggregated data and analysis scripts are available from the Dryad public archive (http://datadryad.org) under accession number doi:10.5061/dryad.kt69d.

## Additional files

Additional file 1: Figure S1. Photographs of the experiments conducted in Atlit and Hohenheim. A) Atlit control, B) Atlit drought, C) Hohenheim control, and D) Hohenheim drought. Additional file 2: Table S1. List of sequence data used in this study. Additional file 3: Table S2. List of differentially expressed genes in response to drought stress common to all accessions. Additional file 4: Table S3. List of candidate genes associated with tolerant response to drought stress. Additional file 5: Table S4. List of candidate genes associated with sensitive response to drought stress. Additional file 6: Table S5. List of gene ontology terms and description found for each of the four accessions. Additional file 7: Figure S2. Zoom-in on differentially expressed alleles associated with drought tolerance. The figure was generated in integrative genomic viewer browser [77] for differentially expressed allele in MLOC_67950.1 (morex_contig_53956).

## Abbreviations

RS: Reproductive success
SD: Standard deviation
RBD: Randomized block design
RPKM: Reads per kilobase of exon per million reads mapped
GO: Gene ontology
FDR: False discovery rate
