# Supplementary figures and images for: RNA-Seq analysis identifies genes associated with differential reproductive success under drought-stress in accessions of wild barley Hordeum spontaneum
Source: BMC Plant Biol. 2015 Jun 9;15:134. doi: 10.1186/s12870-015-0528-z (PMC4459662; doi:10.1186/s12870-015-0528-z)

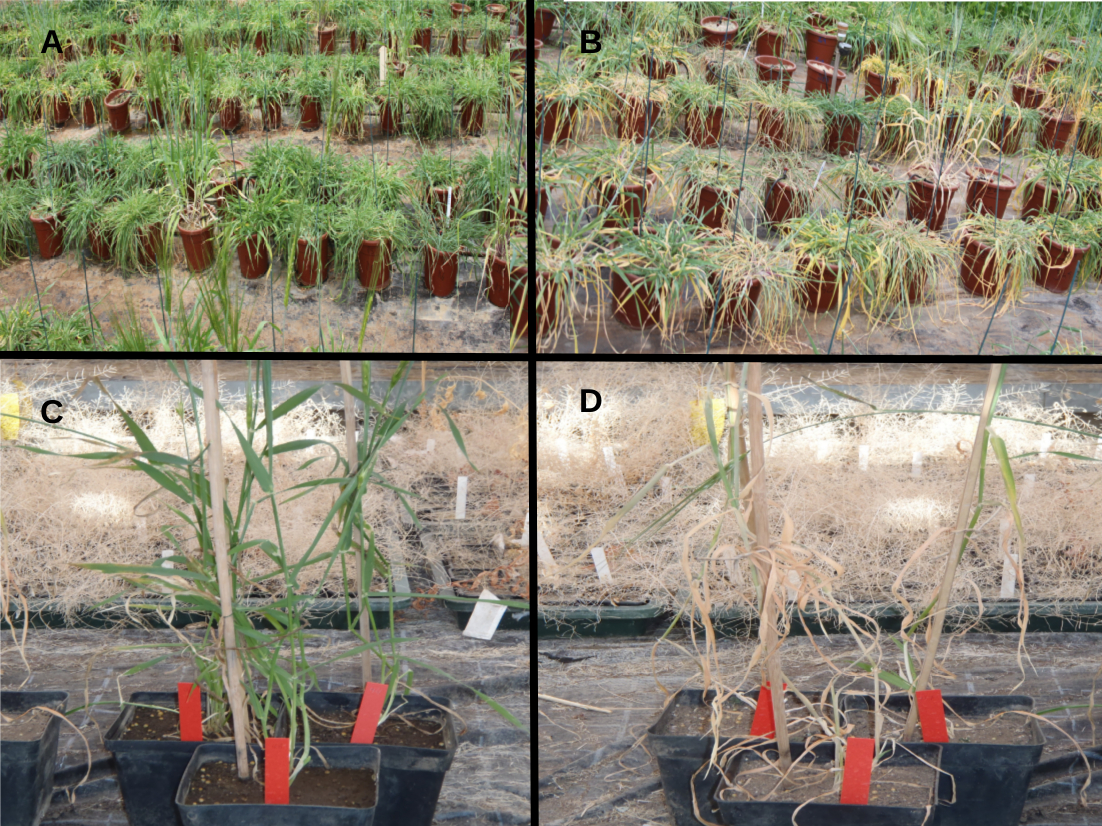

Supplement: Additional file 1: Figure S1. — Photographs of the experiments conducted in Atlit and Hohenheim. A) Atlit control, B) Atlit drought, C) Hohenheim control, and D) Hohenheim drought. [file 12870_2015_528_MOESM1_ESM.jpeg]

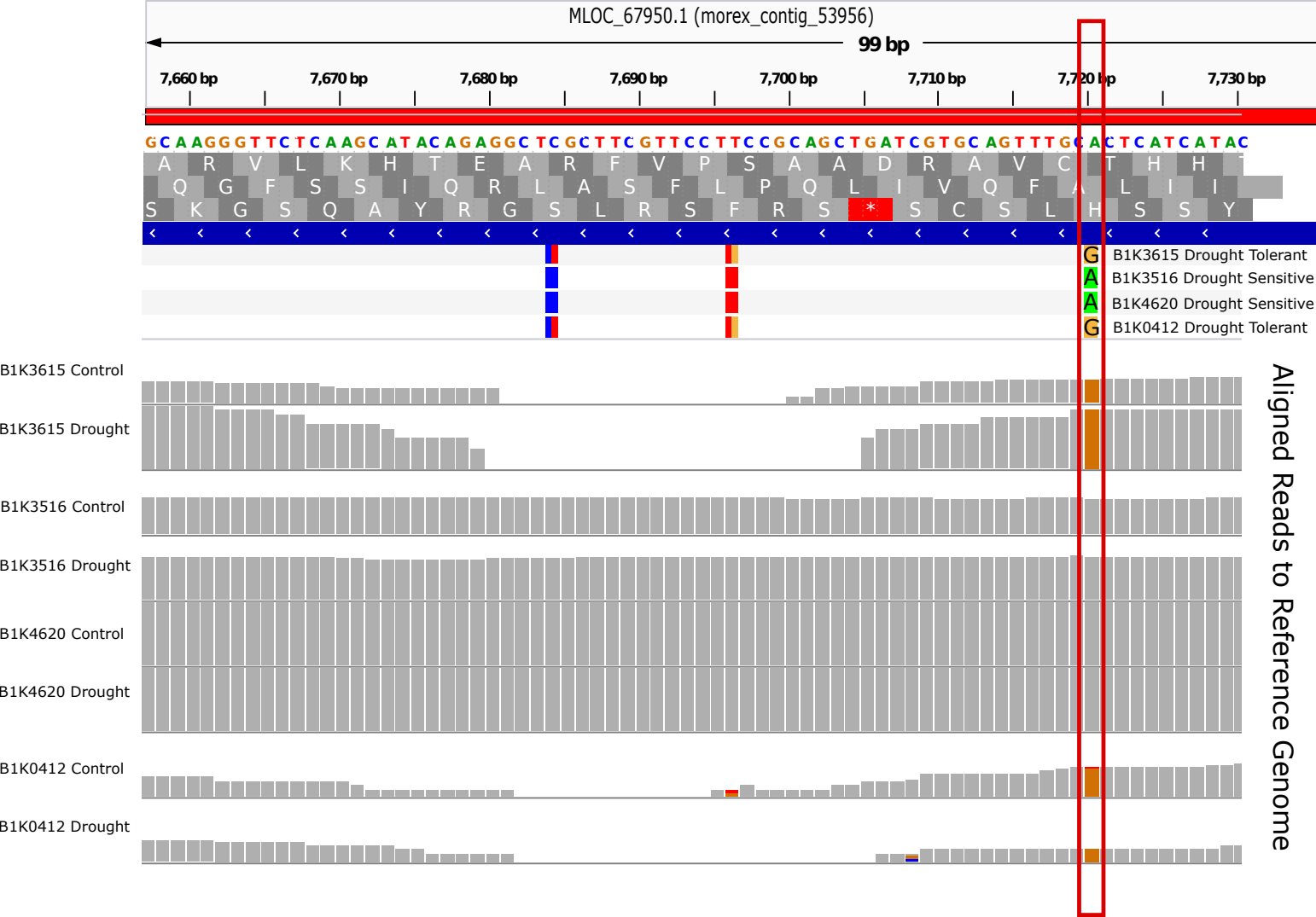

Supplement: Additional file 7: Figure S2. — Zoom-in on differentially expressed alleles associated with drought tolerance. The figure was generated in integrative genomic viewer browser [77] for differentially expressed allele in MLOC_67950.1 (morex_contig_53956). [file 12870_2015_528_MOESM7_ESM.pdf]
